# Supplementary figures and images for: The Combination of PPARα Agonist GW7647 and Imeglimin Has Potent Effects on High-Glucose-Induced Cellular Biological Responses in Human Retinal Pigment Epithelium Cells
Source: Bioengineering (Basel). 2025 Mar 8;12(3):265. doi: 10.3390/bioengineering12030265 (PMC11939608; doi:10.3390/bioengineering12030265)

## Slide 1
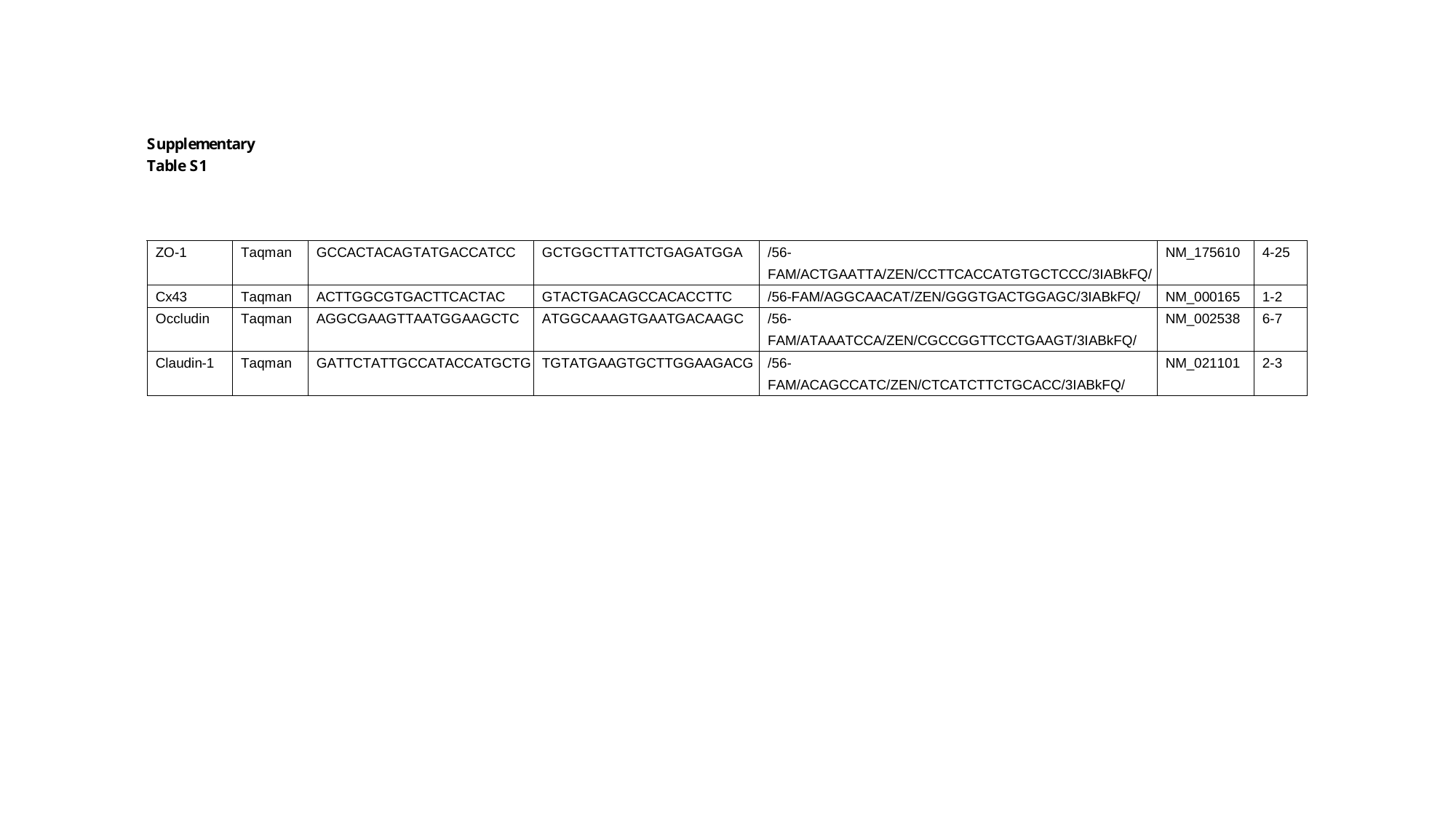

## Slide 2
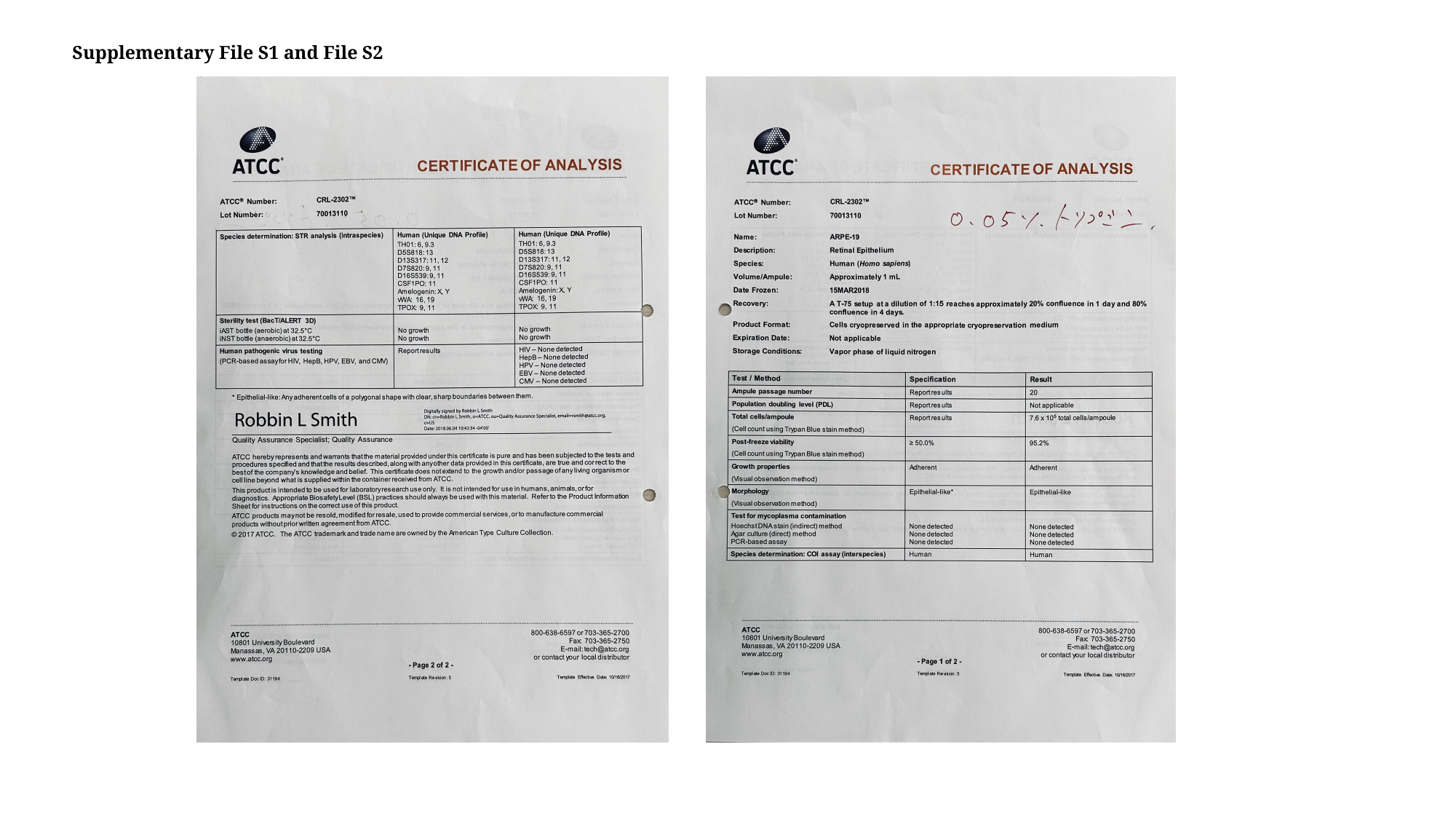

Supplementary File S1 and File S2

Supplement: Supplementary file 1 [file bioengineering-12-00265-s001.zip › bioengineering-3297756-supplementary.pptx]
